# Brain versus Machine Control

**DOI:** 10.1371/journal.pbio.0020430

**Published:** 2004-12-14

**Authors:** Jose M Carmena

## Abstract

Dr. Octopus, the villain of the movie "Spiderman 2", is a fusion of man and machine. Neuroscientist Jose Carmena examines the facts behind this fictional account of a brain- machine interface

“Dr. Octopus,” the villain that terrorizes the city in the most recent film of the popular *Spider-Man* comic, is the ultimate characterization of a brain–machine interface (BMI) on the big screen. In *Spider-Man 2,* the brain is that of nuclear physicist Dr. Otto Octavius, who dreams of harnessing nuclear fusion. The machine is a harness of four mechanical arms designed with tentacle-like flexibility, gripping and vision capabilities, and an artificial intelligence module that gives them some autonomy. The interface between the machine and the brain is at the spinal cord level, with an “inhibitor chip” to prevent the artificial intelligence module in the mechanical arms from taking over Octavius's brain. Controlling this mechanical device with his own thoughts, Octavius is able to manipulate hazardous materials during his fusion experiments. However, things go terribly wrong during the exhibition of one of these experiments: the mechanical arms fuse to Octavius's body while the inhibitor chip is disabled, resulting in the machine gaining partial control of his brain. Unable to subvert the machine to his will and conscience, Octavius, together with the BMI, becomes the villainous Dr. Octopus. At the end of the movie, in a flicker of sanity and heroism, Octavius dramatically sacrifices his life as the only way to terminate the evil machine and save the world.

Although Dr. Octopus is a fictional character, a figment of a vivid imagination, audiences are fascinated by the fact that he is a human BMI. BMIs straddle the worlds of fact and fiction. While the entertainment industry has focused primarily on applications for augmenting cognitive and sensorimotor function, as seen in *Star Trek, Firefox,* and many other science-fiction scenarios, the scientific community has targeted clinical applications, such as neuroprostheses for restoring motor function after traumatic lesion of the central nervous system. The current BMI approach is based on the idea that a human user could enact voluntary motor intentions through a direct interface between his brain and an artificial actuator in virtually the same way that we see, walk, or grab an object with our own natural limbs. Proficient brain control of an external device or actuator should be achievable through training using any combination of visual, tactile, or auditory feedback. As a result of long-term use of the BMI, the brain should be able to “incorporate” (or adapt to) the artificial actuator as an extension of its own body. With these goals in mind, the last five years have witnessed a dramatic increase in BMI-related studies in academic institutions around the world. Subjects have learned to utilize their brain activity for different purposes, ranging from electroencephalogram- and electrocorticographic-based systems (Wolpaw et al. 2002; Leuthardt et al. 2004), in which human subjects control computer cursors, to multielectrode-based systems, in which nonhuman primates control the movements of cursors and robots to perform different kinds of reaching and grasping tasks (Serruya et al. 2002; Taylor et al. 2002; Carmena et al. 2003; Musallam et al. 2004).

These examples of what could be called the first generation of BMIs have something in common: they have been exclusively controlled by neural signals. Even with BMIs that use neural activity recorded with invasive electrodes to yield higher bandwidth and thus allow for the execution of more complex tasks, it remains unclear whether the quality of the signal will ever suffice for a patient to freely, safely, and effectively control a prosthetic arm to perform daily tasks. For instance, the level of motor skill required for dexterous finger manipulation is outstanding. Planning paths and avoiding obstacles while reaching and grasping in unconstrained environments requires similarly fine motor control. Thus, realistic motion through a complex environment with a BMI is extremely challenging and, perhaps, not feasible with the relatively low bandwidth (∼10 Hz) of current BMIs. Even if significant improvements are made in the algorithms used to decode neural activity by, for example, incorporating knowledge from neurophysiological experiments of how motor signals that underlie movements are encoded in the brain, current BMI bandwidth still may not be sufficient to reach the performance level an injured patient would desire.

What does this mean for second-generation BMIs? We may find some inspiration in Dr. Octopus. The fictional BMI in *Spider-Man 2* is innovative in the sense that it is a hybrid system that incorporates both neuronal and artificial control signals. It makes perfect sense to take advantage of the fields of engineering (control theory) and artificial intelligence to build better BMIs—part brain and part robot. In principle, these hybrid BMIs would allow a patient to accomplish a task more efficiently than those relying on neuronal signals alone. For example, in a common task such as reaching for and grasping a glass of water, a hybrid BMI would be fed with both brain and machine control signals; the intention of movement would be decoded directly from neuronal signals, leaving obstacle avoidance and grasping stabilization to the artificial control module of the system. Such a module would get inputs from sensors embedded in the robot, and would produce a control signal that would fuse with the neuronal control signal to augment the final output command.

What ratio of neuronal versus artificial signal would be needed for optimal control of a BMI? In the movie, Octavius's crisis is a severe unbalance in favor of machine control. Science fiction aside, we see the more realistic potential problems of having a physical device gaining autonomous control. Technically, this could be analyzed as too much gain in the artificial control signal, which, in a realistic scenario, would likely result in oscillating behavior, jerky grasping, etc. Hence, safeguarding measures (characterized in the movie as the inhibitor chip in Octavius's brain stem) would be needed to avoid dangerous situations when a chronic neuroprosthesis freely interacts with the real world. For both science and science fiction, the question is the same. Brain and machine: which one gets the power?

## Abbreviations

BMI: brain–machine interface
